# Clinical outcomes of image-guided therapies in patients with adrenocortical carcinoma: a tertiary referral center retrospective study

**DOI:** 10.1093/oncolo/oyae130

**Published:** 2024-06-13

**Authors:** Brenda Chahla, Koustav Pal, Vania Balderrama-Brondani, Feyza Yaylaci, Matthew T Campbell, Rahul A Sheth, Mouhammed Amir Habra

**Affiliations:** Department of Endocrine Neoplasia and Hormonal Disorders, The University of Texas MD Anderson Cancer Center, Houston, TX, United States; Department of Interventional Radiology, The University of Texas MD Anderson Cancer Center, Houston, TX, United States; Department of Endocrine Neoplasia and Hormonal Disorders, The University of Texas MD Anderson Cancer Center, Houston, TX, United States; Department of Endocrine Neoplasia and Hormonal Disorders, The University of Texas MD Anderson Cancer Center, Houston, TX, United States; Department of Genitourinary Medical Oncology, The University of Texas MD Anderson Cancer Center, Houston, TX, United States; Department of Interventional Radiology, The University of Texas MD Anderson Cancer Center, Houston, TX, United States; Department of Endocrine Neoplasia and Hormonal Disorders, The University of Texas MD Anderson Cancer Center, Houston, TX, United States

**Keywords:** adrenocortical carcinoma, ablation, embolization, chemotherapy, survival

## Abstract

**Background:**

Image-guided therapies (IGTs) are commonly used in oncology, but their role in adrenocortical carcinoma (ACC) is not well defined.

**Materials and Methods:**

A retrospective review of patients with ACC treated with IGTs. We assessed response to therapy using RECIST v1.1, time to next line of systemic therapy, disease control rate (DCR), local tumor progression-free survival (LTPFS), and complications of IGTs (based on the Common Terminology Criteria for Adverse Events [CTCAE] version 5.0).

**Results:**

Our cohort included 26 patients (median age 56 years [range 38-76]; *n* = 18 female) who had 51 IGT sessions to treat 86 lesions. IGTs modalities included cryoablation (*n* = 49), microwave ablation (*n* = 21), combined microwave and bland trans-arterial embolization (*n* = 8), bland trans-arterial embolization alone (*n* = 3), radio-embolization (*n* = 3), and radiofrequency ablation (*n* = 2). DCR was 81.4% (70 out of 86), of which 66.3% of tumors showed complete response, 18.6% showed progressive disease, 8.1% showed partial response, and 7.0% showed stable disease. LTPFS rates were 73% and 63% at 1 and 2 years, respectively. Fourteen lesions underwent re-ablation for incomplete response on initial treatment. Sixteen patients (61.5%) received new systemic therapy following IGTs, with a median time to systemic therapy of 12.5 months (95% CI: 8.6 months upper limit not reached). There was 1 reported CTCAE grade 3 adverse event (biloma) following IGT.

**Conclusions:**

IGT use in properly selected patients with ACC is safe and associated with prolonged disease control and delay in the need for systemic therapy.

## Teaser

This study reviewed records of patients with adrenocortical carcinoma treated at a tertiary referral center to evaluate the efficacy and safety of imaging-guided locoregional treatments.

**Implications for practice**

Adrenocortical carcinoma (ACC) is a rare malignancy without effective systemic therapy options. Image-guided therapies (IGTs) can be a safe and efficacious alternative to systemic chemotherapy in properly selected patients. IGTs could lead to prolonged disease control, preserve quality of life, and delay the need for systemic treatment. In our cohort of patients with ACC treated at a tertiary referral center with known experience in ACC management, IGTs were efficacious and safe interventions that helped most patients while having low risk for severe complications.

## Introduction

Adrenocortical carcinoma (ACC) is a rare cancer, with an annual incidence of 1-2 cases per million people.^1^ ACC is slightly more common in women with peak incidence between 30 and 50 years.^1,2^ The stage at presentation is among the most important prognostic factors.^3^ Most patients with localized ACC often develop recurrence after seemingly complete, en-bloc, margin-negative (R0) initial surgical resection, and almost 25% of patients present with metastatic disease.^1,3-7^

Mitotane is the only approved systemic treatment for ACC.^7,8^ However, the response rate to mitotane monotherapy is only 10%-30%.^7^ Thus, in advanced ACC cases, mitotane is often combined with cytotoxic chemotherapy.^7,9^ The most commonly used regimen in advanced/metastatic ACC is a combination of mitotane with etoposide, doxorubicin, and cisplatin (EDP). This regimen has a relatively low response rate (overall response rate of 23%), a high toxicity rate (severe adverse events in more than 50% of cases), and short-lived benefit (median progression-free survival < 6 months).^10^ The role of new treatments, such as immunotherapy and tyrosine kinase inhibitors, in ACC is evolving.^9^

Imaging-guided locoregional treatments (IGTs) include a range of procedures to achieve local tumor control. These procedures include image-guided thermal ablations (such as radiofrequency ablation, microwave ablation, and cryoablation) and trans-arterial embolization (such as chemoembolization and radio-embolization).^3,11^ IGTs are commonly used to achieve local control of many malignancies, but reported experience with IGTs in ACC is limited.^3,9,11-13^ Kimpel o et al. performed a review of the literature and found only 3 articles about trans-arterial embolization, 4 articles on image-guided thermal ablations, and 2 articles on the use of combined IGTs.^11^ Despite limited evidence, current recommendations are in favor of considering IGTs in the treatment of advanced ACC in select patients.^5,14^

We hypothesized that IGTs could delay the need for systemic therapy in ACC by improving local control of treated lesions. To test our hypothesis, we retrospectively reviewed records of patients with ACC treated at a tertiary referral center to evaluate the efficacy and safety of these procedures.

## Methods

### Patients

Institutional review board (IRB; IRB number PA12-0933) approval was obtained for this single institution retrospective study. The study cohort was comprised of 26 ACC patients who underwent IGTs at MD Anderson Cancer Center between January 18, 2006 and April 11, 2023. Inclusion criteria included histopathological confirmation of ACC from either percutaneous biopsy or resection of a primary or metastatic tumor. All patients were cared for by a multidisciplinary team and underwent 1 or more IGT: Cryoablation, microwave ablation, combined microwave and bland trans-arterial embolization, bland trans-arterial embolization alone, radio-embolization, and radiofrequency ablation.

### Safety procedures

Technically challenging procedures were defined as those requiring the use of hydro-dissection, pneumo-dissection, and a wire or balloon to perform ablation while avoiding injury to surrounding vital structures. These procedures are commonly used during the treatment of tumors in technically challenging locations.^15^

### Image interpretation

The efficacy of IGTs was objectively assessed based on Response Evaluation Criteria in Solid Tumors version 1.1 (RECIST 1.1).^16^ Each ablated focus was individually assessed for response, and standardized ablation consensus terminology was followed.^17^ The sum of 2 tumor axis diameters was assessed per RECIST 1.1 criteria before and after IGT.

In assessing the initial postablation cross-sectional imaging study, we defined residual unablated tumors as irregular peripheral or nodular enhancement within the ablated area. Image analysis and response assessment were performed by a trained interventional radiologist (R.A.S.).

### Statistical analysis

We used descriptive statistics to report demographic and tumor characteristics, IGT procedures, complications, and pathologic findings. Survival curves were generated using the Kaplan-Meier product-limit estimator. Overall survival (OS) was measured in months from the date of the first IGT to the date of death or last follow-up. Local tumor progression-free survival (LTPFS) was measured from the date of each IGT to the date when local tumor progression at the ablation site was detected on cross-sectional imaging. The estimated chemotherapy-free survival was measured in months from the date of the first IGT to the date of the next line of systemic therapy^18^; this analysis only included the patients who received systemic therapy starting 30 days after IGT. The cumulative incidence function was used to evaluate and visualize the probability of local tumor progression with death as a competing risk.

Disease control rate (DCR) was calculated as all tumors which had either stable disease, partial response, or complete response on RECIST 1.1 post-ablation assessment.^16^

Cox proportional hazards modeling was performed to identify variables associated with local tumor progression. The variables included in the univariable analysis were number of ablation needles, technically challenging procedures, Weiss score, Ki-67 value, resection status, surgical approach for the primary tumor (laparoscopic vs open), and tumor size.

Complications were categorized based on the Common Terminology Criteria for Adverse Events, version 5.0.^19^ A *P*–value <.05 was considered statistically significant in all analyses. Statistical analysis was performed with R software version 4.2.3 (R Foundation for Statistical Computing, Vienna, Austria).

## Results

### Patient characteristics

**Figure 1.**
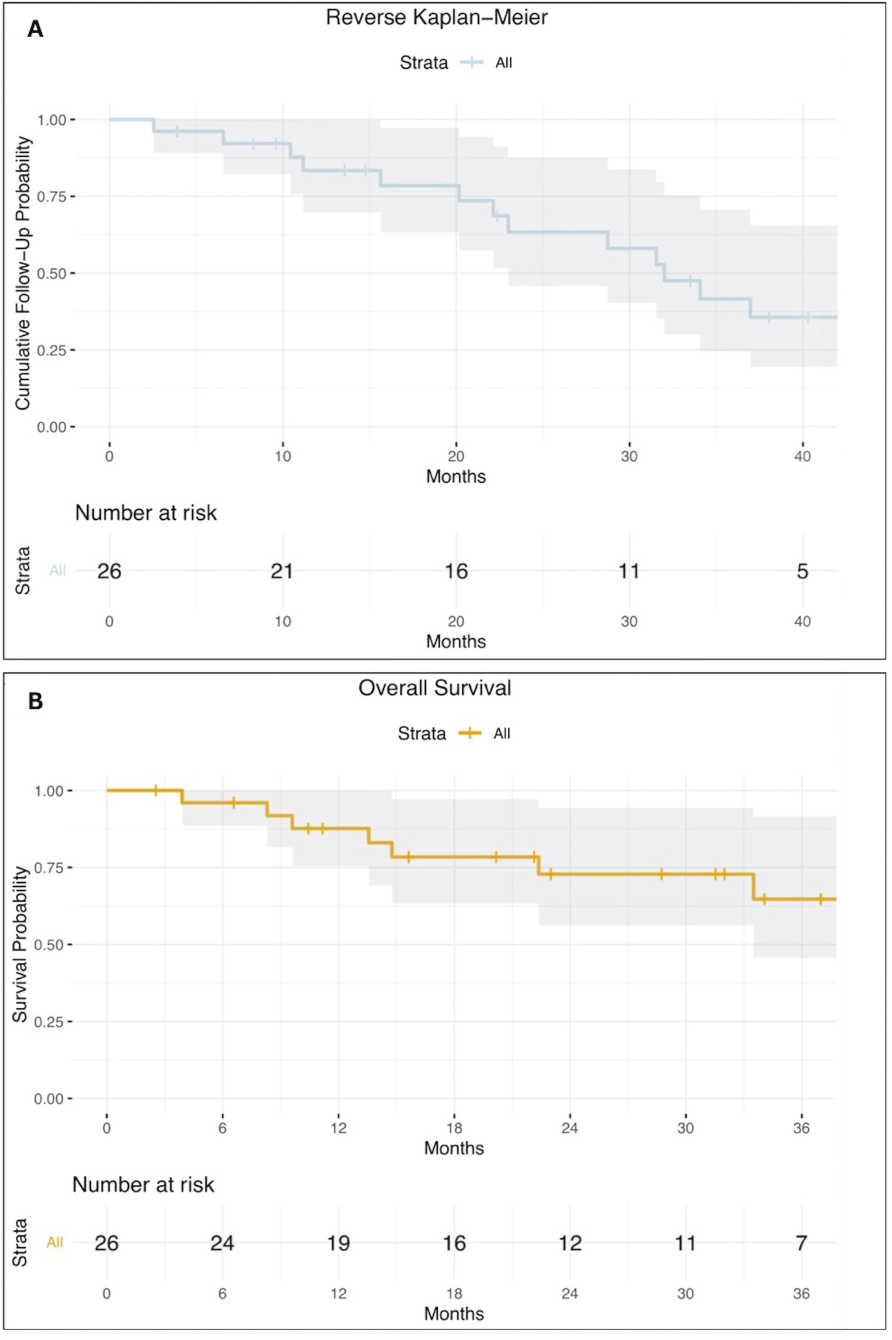
(A) The reverse Kaplan-Meier curve was used to estimate the overall follow-up time of the study population. The median follow-up time was calculated to be 32 months (95% CI: lower 22 months, upper not reached). The × axis represents time, while the y-axis shows the cumulative follow-up probability. (B) The Kaplan-Meier curve depicts the overall survival of the study population over 3 years. The x-axis represents time, while the y-axis shows the survival probability. OS was 83% (95% CI: 75%-100%) at 1 year and 72% (95% CI: 56%-94%) at 2 years.

Our cohort included 26 patients with pathologically confirmed ACC. The median Ki-67 value was 20% (range: 2%-79%), and the median Weiss score was 6 (range: 3-9). The surgical approach for the primary tumor consisted of 22 open surgeries and 4 laparoscopic resections (Table 1). The median follow-up time from the time of IGT was calculated using the reverse Kaplan-Meier estimator and it was 32 months (95% CI: lower 22 months, upper not reached; Figure 1A).

**Table 1.** Patient characteristics.

Characteristics	No. (%)
Age at ACC diagnosis, median (range)	56 (38-76)
Ki-67, median (range)	20 (2-79)
Weiss score, median (range)	6 (3-9)
Sex	
Female	18 (69.2)
Male	8 (30.8)
Mode of surgery	
Open	22 (84.6)
Laparoscopic	4 (15.4)
Resection status	
R0	18 (69.2)
R1	2 (7.7)
R2	2 (7.7)
Rx	4 (14.4)
Functional status	
Nonfunctional	9 (34.6)
Hormonally active	15 (57.7)
N/A	2 (7.7)
Prior systemic therapy (before first IGT), no. (%)	
Mitotane	13 (50.0)
Systemic chemotherapy	10 (38.5)
Immunotherapy	4 (15.4)
Other	3 (11.5)
Concurrent systemic therapy (concurrent with first IGT), no. (%)	
Mitotane	6 (23.1)
Immunotherapy	5 (19.2)
Systemic chemotherapy	3 (11.5)
Other	0 (0.0)

Abbreviation: acc, adrenocortical carcinoma.

### Procedures

A total of 51 unique IGT procedures were performed for 86 treated lesions. Different minimally invasive interventions were performed for a wide variety of anatomical locations. These included the adrenalectomy bed, liver, intraperitoneal sites, lungs, lymph nodes, retroperitoneal soft tissues, and other soft tissues. The following IGT modalities were used Cryoablation, microwave ablation, combined microwave ablation and bland trans-arterial embolization, bland trans-arterial embolization, radio-embolization, and radiofrequency ablation (Table 2). Fourteen tumors were re-ablated once (16.3%). Of these 14 re-ablated lesions, 2(14.3%) had a consequent complete response, 9 (64.3%) had progressive disease, and 3 (21.4%) had stable disease or partial response.

**Table 2.** Image-guided therapies performed and outcomes (*N* = 26 patients, 51 procedures, 86 treated lesions).

Number of IGT procedures performed per patient, median (range)	1 (1-4)
Number of lesions ablated per patient, median (range)	2 (1-9)
Procedure performed, no. (%)	
Cryoablation	49 (57.0)
Microwave	21 (24.4)
Microwave, bland trans-arterial embolization combined	8 (9.3)
Bland trans-arterial embolization	3 (3.5)
Radio-embolization	3 (3.5)
Radiofrequency ablation	2 (2.3)
Treated site, no. (%)	
Liver	37(43.0)
Intraperitoneal	11(12.8)
Adrenalectomy bed	10 (11.6)
Lung	6 (7.0)
Lymph node	6(7.0)
Retroperitoneal soft tissue	6(7.0)
Soft tissue (elsewhere)	10(11.6)
Re-ablated tumors, no. (%)	14 (16.3)
Largest pre-IGT anteroposterior tumor dimension, median (range)	2.3 cm (0.5-8.6)
Response to treatment, no. (%)	
Complete response	57 (66.3)
Progressive disease	16 (18.6)
Partial response	7 (8.1)
Stable disease	6 (7.0)
Local tumor progression, no. (%)	25 (29.1)
Safety procedures performed, no. (%)	26 (30.2)
Type of safety procedure done, no. (%)	
Hydro-dissection	22 (84.7)
Chest tube (artificial pneumothorax)	2 (7.7)
Coons wire, balloon	1 (3.8)
Pneumo-dissection	1 (3.8)
Next-line systemic therapy (after first IGT), no. (%)	
Yes	16 (61.5)
No	10 (38.5)
Next-line systemic therapy (after first IGT), no. (%)	
Systemic chemotherapy	7 (26.9)
Immunotherapy	6 (23.1)
Mitotane, immunotherapy combined	1 (3.8)
Other	2 (7.7)
Status on last follow-up, no. (%)	
Alive with disease	12 (46.1)
Alive without disease	4 (15.4)
Dead	10 (38.5)

Abbreviation: igt, image-guided locoregional treatment.

The median pre-IGT anteroposterior tumor size was 2.3 cm (range: 0.5-8.6), the median transverse size was 1.7 cm (range: 0.4-11.8), and the combined diameters were 4.0 cm (range: 0.9-20.4; Table 2).

### Long-term outcomes after ablation

In response to IGT, the overall DCR was 81.4% (70 out of 86), of which 66.3% of tumors showed complete response, 18.6% showed progressive disease, 8.1% showed partial response, and 7.0% showed stable disease (Table 2).

Of 86 lesions, only 25 (29.1%) had local tumor progression. LTPFS rates were 73% and 63% at 1 and 2 years, respectively. OS rates were 83% and 72%, at 1, and 2 years, respectively (Figures 1B and 3). The median OS and LTPFS were not reached.

**Figure 2.**
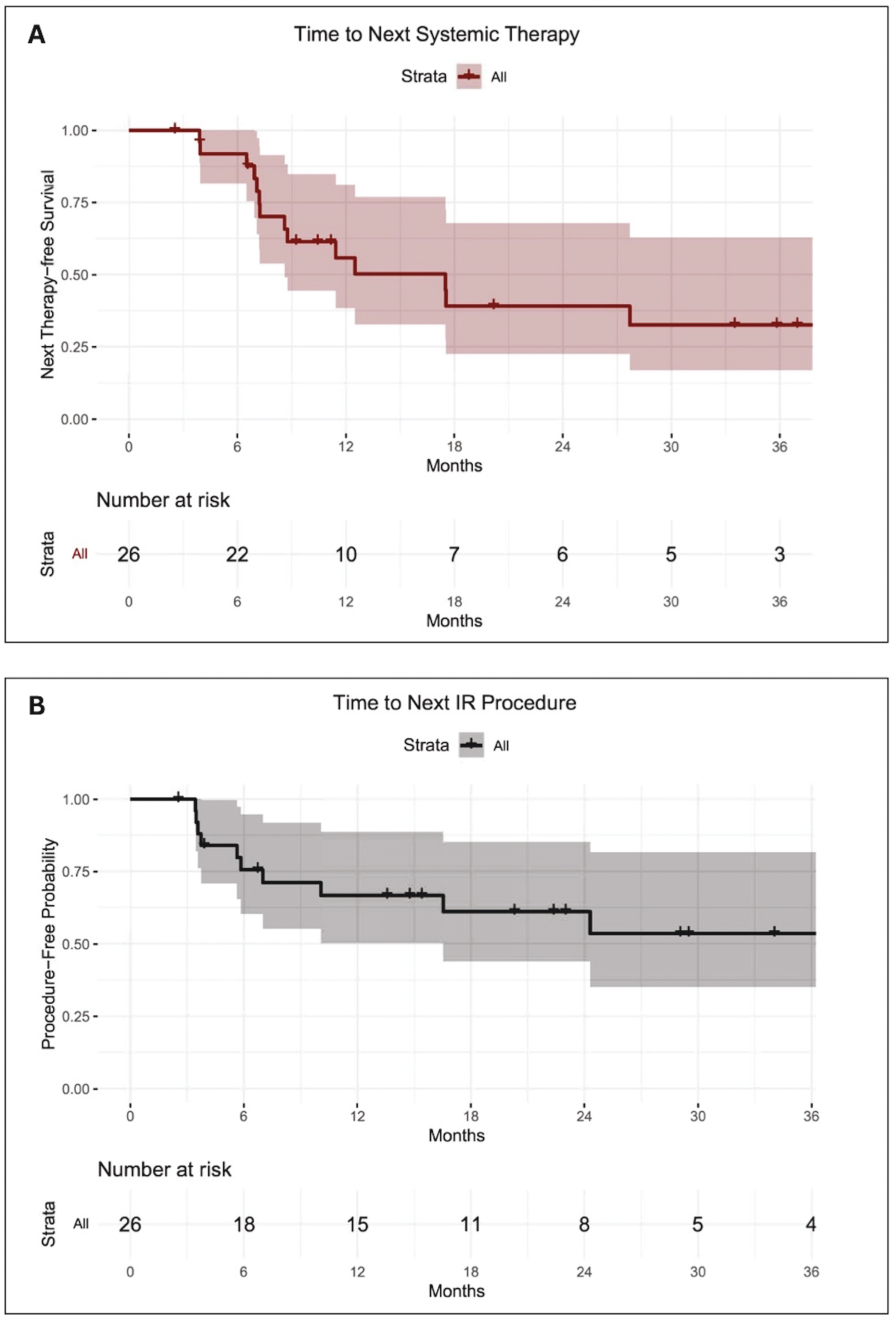
(A) This graph illustrates the time to the next systemic therapy in the study population. The x-axis represents time, and the y-axis denotes the probability of remaining free from subsequent systemic treatment. The median time to the next line of systemic therapy is 12.5 months (95% CI: 8.6 months upper limit not reached). (B) This graph displays the time of the patient’s next interventional radiology (IR) procedure. The *x*-axis represents time, and the y-axis denotes the probability of remaining free from subsequent IR intervention. The median time for the next IR therapy is 28 months.

Sixteen (61.5%) patients received systemic therapy after their first IGT. Chemotherapy was given after 7 procedures, immunotherapy alone after 6 procedures, combination of immunotherapy and mitotane after 1 procedure, and other forms of systemic therapy were given after 2 procedures (Table 2). The median time to the next line of systemic therapy was 12.5 months (95% CI: 8.6 months upper limit not reached) (Figure 2A). A sub-group analysis for patients on concurrent systemic therapy during IGT (*n* = 10) and those not receiving any systemic therapy at time of IGT (*n* = 16) was performed. There was no significant difference in time to next line of systemic therapy and local tumor progression between both groups (*P* = .68 and 0.65, respectively). The estimated median time to next IGT procedure was approximately 24 months (53.5%, [95% CI 10 months to upper not reached within the study period]; Figure 2B).

### Complications

The overall complication rate was 7.8% (4 out of 51 procedures). Only 1 of these complications was grade 3 or higher, where a biloma formed 5 months after the procedure. It is important to note that less than 1 month after the IGT, this patient underwent a laparoscopic cholecystectomy with sphincterotomy for cholecystitis, which could be by itself a cause for the biloma.^20,21^ The other 3 complications included 2 grade 1 stable hematomas, and 1 grade 2 pneumothorax which required a chest tube that was removed after more than 2 days.

### Regression analysis

In our statistical analysis, Cox proportional hazards regression was performed for the following variates for local tumor progression: Tumor size, technically challenging procedures, number of probes used (if ablation was done), Ki-67 value, Weiss score, R0 resection status, and whether laparoscopic or open surgery was performed for resection of the primary tumor (Table 3). Only technically challenging ancillary procedures were significantly associated with progression, with a hazard ratio of 2.87 (1.26-6.41, *P *= .01; Table 3).

**Table 3.** Univariable Cox proportional hazards model results.

Variate	Hazard ratio	Lower CI	Upper CI	*P*
Probes	0.89	0.55	1.46	0.66
Technically challenging ancillary procedures	2.87	1.29	6.41	0.01
Weiss score	1.47	0.93	2.31	0.1
Ki-67	1.01	0.99	1.03	0.17
R0 resection	4.07	0.96	17.28	0.06
Laparoscopic surgery	0.83	0.19	3.55	0.8
Open surgery	1.2	0.28	5.13	0.8
Tumor size (RECIST 1.1 measurement)	0.99	0.87	1.12	0.84

Abbreviation: recist, response evaluation criteria in solid tumors.

**Figure 3.**
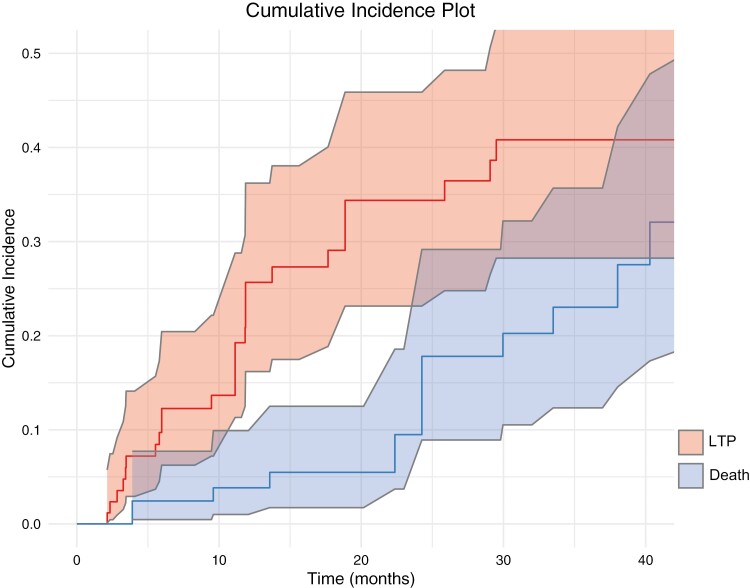
This graph depicts the cumulative incidence of local tumor progression and death as a competing event over time. The cumulative incidence function was performed to account for death as a competing event. At 20 months, the cumulative incidence of experiencing local tumor progression was 34.4% (95% CI: 23%-46%). The cumulative incidence of death was 5.5% (95% CI: 1.7%-12.5%).

The cumulative incidence function was performed to account for death as a competing event. At 20 months, the cumulative incidence of experiencing local tumor progression was 34.4% (95% CI: 23%-46%). The cumulative incidence of death was 5.5% (95% CI: 1.7%-12.5%; Figure 3).

## Discussion

**Figure 4.**
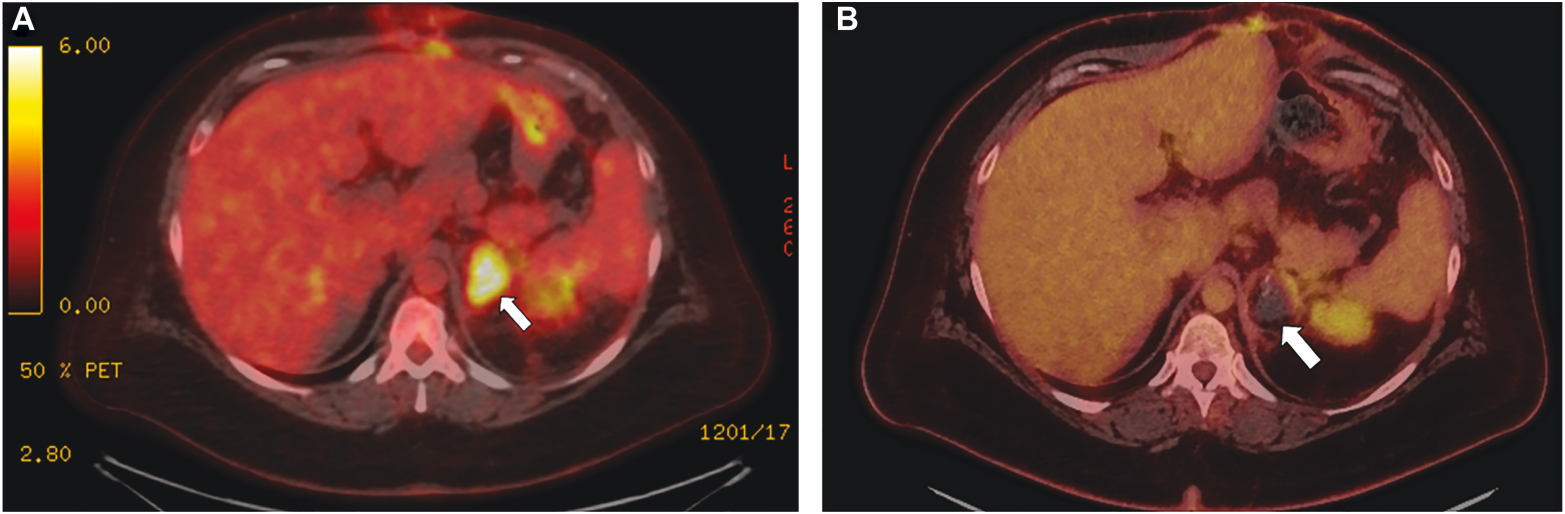
Positron emission tomography scans of a patient who was diagnosed with left adrenal adrenocortical carcinoma. (A) An FDG-avid tumor in the left adrenal gland was resected using left open adrenalectomy with R2 resection and shows FDG uptake in the left adrenal remnants. (B) Subsequent 3-month follow-up demonstrates no FDG uptake, showing complete response to cryoablation.

Our findings support our hypothesis that IGTs can improve local control of treated lesions in patients with ACC and potentially preserve quality of life by delaying the need to initiate or transition to the next line of systemic therapy. In our study, patients with ACC who underwent IGT alone or combined with other systemic treatments showed comparable results to patients in other studies.^3^ The median follow-up time from the time of the first IGT in our study was calculated to be 32 months. This number is comparable to the median follow-up time of 26.5 months found in previous studies assessing thermal ablations only.^11^ Among the tumors treated in our study, 66.3% had complete response after IGT procedures, 8.1% showed partial response, and 7.0% showed stable disease. DCR was calculated to be 81.4% (70 out of 86). These findings further support previous studies that assessed the role of IGT in treating ACC.^3,12,13,22^ In fact, a study assessed the role of transcatheter arterial chemoembolization of 103 metastatic ACC lesions in the liver.^23^ Responses were assessed using RECIST and 22% showed partial response, 65% showed stable disease and 13% had progressive disease.^23^ Added to that, Roux et al showed similar results with a DCR of 63% (19 out of 30) and 85% (17 out of 20) for patients who underwent thermal ablations and trans-arterial embolizations, respectively.^11,24^Figure 4 illustrates the case of one of our patients who had a complete response to cryoablation (Figure 4).

In this cohort of patients who had IGTs, the OS rate at 2 years was 72% which is higher than our previously published experience with patients with stage IV disease who had a 2-year OS rate of 24% in patients seen between 1998 and 2007 and 46% in more recent patients seen between 2007 and 2019.^4^ The 2-year OS rate was 63% for patients with stages I-III ACC who had recurrence and 34% for stage IV patients who did not receive IGTs (our unpublished data). The median OS and LTPFS were not reached. The median time to next line of systemic therapy was 12.5 months and the median time to next IGT procedure was approximately 24 months. These clinical implications suggest that IGT is a reasonable alternative to surgery or systemic therapy in highly selected patients with metastatic ACC because of the shorter recovery time, the lower risk of comorbidity, and the possibility of performing repeat interventions if local progression happens. We also performed a sub-group analysis for patients on concurrent systemic therapy during IGT and those not receiving any systemic therapy at time of IGT. We found no significant difference in time to next line of systemic therapy and local tumor progression between both groups. Mauda-Havakuk et al reported similar results with a 2- and 5-year OS rates of 84.5% and 51%, respectively.^3^

Mauda-Havakuk et al also reported that the use of a combination of IGTs, chemotherapy, and surgery in patients with advanced ACC was associated with longer 2- and 5-year survival in comparison to the use of surgery and chemotherapy alone or chemotherapy and radiation therapy alone.^3,11^ As previously discussed, the systemic treatments for ACC are few, and the available treatment options have shown limited efficacy, especially in patients with advanced disease.^2,25,26^ Additionally, these treatments are associated with many adverse effects.^2,26,27^ This suggests that patients treated with IGT may have a beneficial treatment holiday while avoiding the side effects of systemic treatment and prolonging OS and LTPFS. IGTs are usually considered in patients with ACC who are not candidates for surgical resection or in patients with progression despite surgical resection and systemic therapy.^1-3,5-10,12,22,28^ Despite the importance of surgery, most patients with ACC present with locally advanced (34% with stage 3) or metastatic disease (26% with stage 4) and are not candidates for curative surgical resection.^2,29^ Combining multiple treatment options in these patients, including surgery, systemic chemotherapy, trans-arterial chemoembolization, and radiofrequency ablation, is recommended.^2^ In fact, metastasectomy by ablation or resection improved survival in patients with ACC and certain breast, lung, and colon cancers.^30-32^ A retrospective study assessing the feasibility and safety of cryoablation in 40 adrenal metastases from several different primary sites showed that cryoablation was a safe, effective, and low-morbidity alternative for surgical resection.^15^ However, this study assessed adrenal metastasis in general and not ACC specifically, and more studies are needed to study the safety of IGT in ACC.

Additionally, previous studies on a few patients found radiofrequency ablation to be safe and effective in treating metastatic ACC when the lesions are less than 5 cm in diameter.^12,13^ A study performed by Cazejust et al on patients with advanced ACC treated with trans-arterial chemoembolization found that higher response rates were observed in lesions with a diameter of less than 3 cm.^11,23^ Our population’s median combined AP and transverse tumor size was 4.0 cm (0.9-20.4). Moreover, our analysis found that tumor size was not significantly associated with local tumor progression, suggesting that tumor size may not play a role in LTPFS.

Interestingly, technically challenging procedures affected the LTPFS in a statistically significant manner (*P = *.001). Technically challenging ancillary procedures involve the use of tissue displacement methods to safely separate vital structures such as the bowel, stomach, or ureters from the target lesion.^15^ These procedures were implemented in 22 (25.6%) of the treated lesions in our study. For these specific tumors, margins for complete ablation may be difficult to assess or achieve in a single ablation procedure, and this should be accounted for in the initial planning of IGT.

IGTs are localized treatment options with minimal complications.^3,22^ Veltri et al discussed the radiofrequency and microwave ablation of 32 lung and liver metastases from ACC, and they reported only 1 major adverse event (intrahepatic hematoma with subsequent right hemothorax).^22^ Roux et al reported four grade 3 adverse events (2 bleedings, 1 gastrointestinal fistula, and 1 mild pancreatitis) and three grade 4 adverse events (2 adrenal insufficiencies and 1 pleural hemorrhage).^24^ Mauda-Havakuk et al described 2 grade 3 or higher adverse events (1 hematoma and 1 transient atrial fibrillation and electrolytes imbalance).^3^ Wood et al only reported 1 patient with a multimicrobial abscess 11 weeks after his third radiofrequency ablation session.^12^ These findings are consistent with our results. Only 4 of the 51 procedures performed for our patients had complications, only 1 of which was a grade 3 complication (biloma that developed 5 months after the procedure). This biloma might also be attributed to a laparoscopic cholecystectomy with sphincterotomy that the patient underwent less than a month after the IGT. In fact, the formation of a biloma was previously reported in several cases as a complication of sphincterotomy.^20,21^ The other 3 complications included 2 grade 1 stable hematomas, and 1 grade 2 pneumothorax which required a chest tube that was removed after more than 2 days.

Limitations of our study include its retrospective nature and the small sample size. Moreover, a longer follow-up time would be needed to effectively assess the long-term outcomes of IGT. The absence of a control group might have also affected our interpretation of the results. However, since ACC is very rare with a poor prognosis, a large sample size and a control group would be difficult to implement. Hence, small retrospective studies are important. More studies assessing local tumor progression rates based on the stage of the disease and tumor size might be needed in the future.

## Conclusion

IGTs have shown promising efficacy and safety profiles in the treatment of select patients with ACC. IGT use was associated with prolonged disease control in some patients and delayed the need for systemic therapy. These findings hold promise as ACC has a generally poor prognosis with limited treatment options.

## Data Availability

The data underlying this article will be shared on reasonable request to the corresponding author.
